# Ultrasonication-Assisted Solvent Extraction of Quercetin Glycosides from ‘Idared’ Apple Peels

**DOI:** 10.3390/molecules16129783

**Published:** 2011-11-25

**Authors:** H. P. Vasantha Rupasinghe, Priya Kathirvel, Gwendolyn M. Huber

**Affiliations:** Tree Fruit Bio-product Research Program, Department of Environmental Sciences, Nova Scotia Agricultural College, P.O. Box 550, Truro, Nova Scotia, B2N 5E3, Canada

**Keywords:** flavonols, extraction, ultrasonication, quercetin, quercetin glycoside, acidification, apple

## Abstract

Quercetin and quercetin glycosides are physiologically active flavonol molecules that have been attributed numerous health benefits. Recovery of such molecules from plant matrices depends on a variety of factors including polarity of the extraction solvent. Among the solvents of a wide range of dielectric constants, methanol recovered the most quercetin and its glycosides from dehydrated ‘Idared’ apple peels. When ultrasonication was employed to facilitate the extraction, exposure of 15 min of ultrasound wavelengths of dehydrated apple peel powder in 80% to 100% (v/v) methanol in 1:50 (w:v) solid to solvent ratio provided the optimum extraction conditions for quercetin and its glycosides. Acidification of extraction solvent with 0.1% (v/v) or higher concentrations of HCl led to hydrolysis of naturally occurring quercetin glycosides into the aglycone as an extraction artifact.

## 1. Introduction

Dietary plant polyphenolic molecules, specifically flavonoids, have become of a great interest due to their widely reported potential health benefits [1,2,3,4]. Quercetin is one of the most studied plant flavonoids and has been reported to have antioxidant, anticarcinogenic, antiinflammatory, antiaggregatory, antihypertensive and neuroprotective effects [5,6,7,8]. Quercetin glycosides have also been reported to be the most common flavonol in diet due to their presence in frequently consumed foods such as apples, onions and tea [5,9].

In plant tissues, flavonoids, including quercetin, are usually found in conjugated forms with sugars such as glucose, galactose and rhamnose [9,10,11]. These glycosylated quercetins are differentially distributed in the fruit tissue. For example, quercetin glycosides are found almost exclusively in the peel of apples [9,12,13,14]. There are a number of factors that influence flavonoid extraction from the plant matrix such as chemical nature of extraction solvent, sample to solvent ratio, sample particle size, disruption techniques, temperature as well as time of exposure [15]. Various methods for the extraction and quantification of phenolic molecules from apples have been reported [9,14,15,16,17,18].

In the past few years, there has been an increasing use of ultrasonication as a method to facilitate and accelerate extraction of phenolics from plant matrices [19]. This technique is particularly effective compared to other conventional extraction methods because it allows the disruption of cell compartments facilitating the interaction of phenolic molecules with solvents at reasonably low temperatures [20]. Moreover, other advantages include, drastically reduced processing time; consumption of less energy; increased recovery; and reduced thermal degradation effects [21].

The literature reveals that there is no extraction procedure that is universally suitable for structurally diverse phenolic molecules due to their differing chemical properties such as solubility, degree of polymerization and interaction with other components of the plant matrix [15]. This warrants standardizing extraction procedures for specific sub-classes of phenolic molecules of interest. Apple peels, one of the major dietary source for flavonols, *i.e.*, quercetins [9,22], are also a by-product of apple processing industry [23,24]. Previous research also indicates strong radical scavenging and antioxidative protection property of polyunsaturated fatty acid oxidation of polyphenolic extracts of apple peels [24,25]. Hence, investigation of the optimization of extraction procedures for recovery of quercetin and its major glycosides from apple peels is useful. The purpose of this research was to compare the efficiency of solvents of varying polarities for the extraction of quercetin and its glycosides from dehydrated apple peel powder and then to optimize the extraction of flavonols using a selected solvent under ultrasonication-assisted extraction procedure. The effect of acidifying the extraction solvent was also examined.

## 2. Results

### 2.1. Effect of Extraction Solvents of Varying Polarity

The efficacy of solvents in flavonol extraction followed the order: Methanol > acetone > ethyl acetate (Table 1). Highly polar (water) and non-polar (chloroform) solvents were not effective in extracting flavonols from apple peels. Methanol was chosen for further studies as it provided the highest recovery of flavonols.

**Table 1:** Effect of extraction solvents of varying polarity on the recovery of selected flavonols from dehydrated apple peels *^a^*.

Solvent	Flavonols (mg/100 g DW)					
	Dielectric Constant	Quercetin	Quercetin-3- *O*-galactoside	Quercetin-3- *O*-rhamnoside	Quercetin-3- *O*-rutinoside	Total Quercetin & Glycosides
Water	80	ND	ND	ND	ND	ND
Methanol	33	1.3 ^a^	64.6 ^a^	26.5 ^a^	2.2 ^a^	94.6 ^a^
Acetone	20.07	0.9 ^a^	57.5 ^b^	19.9 ^b^	1.0 ^a^	79.3 ^b^
E. acetate	6.02	ND	12.3 ^c^	8.8 ^c^	ND	21.1 ^c^
Chloroform	4.72	ND	ND	ND	ND	ND

ND-Not detected. *^a^* Means followed by the same letter within each row are not significantly different [Tukey’s Studentized range test (P < 0.05)].

### 2.2. Effect of Different Concentrations of Methanol and Extraction Time

**Figure 1:**
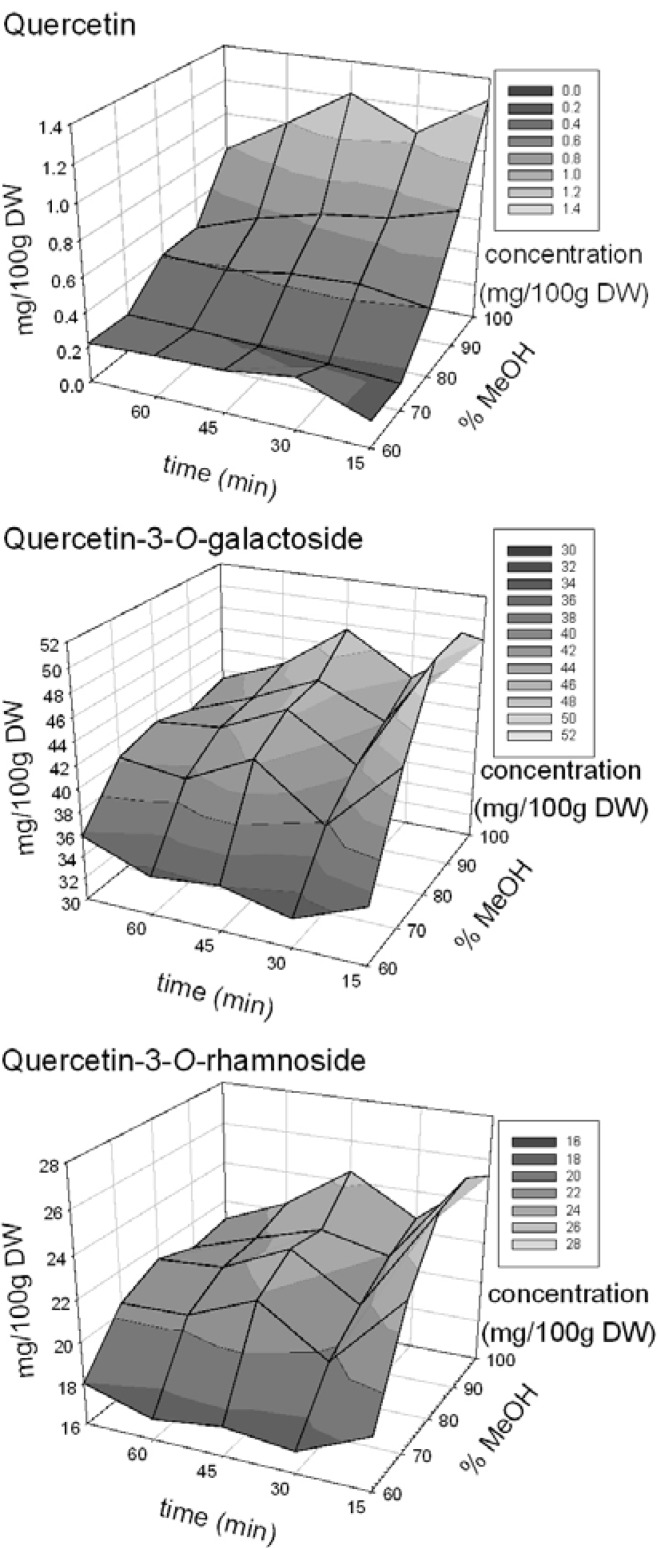
Response surface plots indicating the extraction of flavonols (mg/100 g DW) using various concentrations of methanol (%) at different ultrasonic bath times (min).

The response surface plots for the flavonols reflect the interaction effects (methanol × extraction time) through visible curvatures (Figure 1). The interaction between methanol concentration and time in the ultrasonic bath was significant for quercetin, quercetin-3-*O*-galactoside and quercetin-3-*O*-rhamnoside (p < 0.05). The interaction as well as the main effects was not found to be significant for quercetin-3-*O*-rutinoside, which could be due to the very low concentrations of this molecule recovered from apple peels.

The response surface curves for quercetin-3-*O*-galactoside and quercetin-3-*O*-rhamnoside, were very similar in shape (Figure 1). Both graphs showed a sharp increase in the recovery of the molecules from 60% to 70% methanol, then a slower increase from 80% to 100% aqueous methanol, for all time periods. However, the shape of the quercetin response curve is different from the glycosides, in that the recovery of the aglycone showed a more consistent increase from 60% to 100% methanol. An analysis of variance, blocked by flavonol, was performed to determine if the recovery of flavonols differed among the extraction solvents of aqueous methanol at 15 min. The Tukey’s mean comparison revealed that there was no significant difference in concentration of flavonols extracted using 80% *vs.* 100% methanol as well as 70% *vs.* 80% methanol; however, 60% methanol extracted the lowest concentration of flavonols. In terms of sonication time, there was a general decrease in molecule recovery from 15 min to 75 min and an analysis of variance with Tukey’s means comparison revealed a steady decline in extracted flavonols with 100% methanol after 15 min of ultrasonication (p = 0.0001).

### 2.3. Effect of Acidification of Methanol Using Hydrochloric Acid

The recovery of the selected flavonols in response to acidification of methanol ranged from 41.3 to 97.4 mg per 100 g DW (Table 2). The recovery of the molecules showed an interesting trend such that increasing concentration of HCl was associated with increasing concentration of quercetin aglycone and a decrease in the galactoside and rhamnoside concentrations (Table 2). Furthermore, a significantly lower concentration of quercetin and glycosides were obtained at higher levels of acidification (1.0 and 2.0% HCl in methanol).

**Table 2:** Distribution of quercetin and selected quercetin glycosides in apple peel extracted with methanol containing various concentrations of hydrochloric acid *^a^*.

% HCl in Methanol	Quercetin	Quercetin-3- *O*-galactoside	Quercetin-3- *O*-rhamnoside	Quercetin-3- *O*-rutinoside	Total quercetin and glycosides (mg/100 g DW)
	(mg/100 g DW)	(mg/100 g DW)	(mg/100 g DW)	(mg/100 g DW)	
0	1.48 ^c^	63.5 ^a^	23.8 ^a^	1.35 ^a^	90.1 ^ab^
0.01	1.43 ^c^	61.9 ^a^	23.6 ^a^	1.18 ^ab^	88.1 ^b^
0.1	24.7 ^b^	55.4 ^b^	15.9 ^b^	1.49 ^ab^	97.4 ^a^
1.0	36.3 ^a^	12.4 ^c^	0.51 ^c^	1.15 ^ab^	50.4 ^c^
2.0	34.4 ^a^	5.48 ^d^	0.58 ^c^	0.89 ^b^	41.3 ^c^

*^a^* Means followed by the same letter within each row are not significantly different [Tukey’s Studentized range test (P < 0.05)].

## 3. Discussion

Understanding the distribution and concentration of bioactive flavonol molecules in dietary sources is important for both fundamental researches in natural product chemistry as well as development of natural health products. Discrepancies in the reported concentrations of flavonols from the dietary sources could be due to a variety of factors. However, standardization of extraction procedures for specific phenolic sub-classes is one of the important requirements. Currently, most of the reported phenolic extraction procedures from plant sources are arbitrarily selected methods without optimization for specific molecules of interest. As well, one extraction method has been commonly used for quantification of many sub-classes of phenolics due to the convenience. This study was an attempt to optimize the recovery of selected flavonols, quercetin and its glycosides, from dehydrated and ground ‘Idared’ apple peels using an extraction procedure based on methanol and ultrasonication.

A comparative study of extraction solvents of varying polarity revealed that methanol was effective in extracting quercetin and quercetin glycosides from dehydrated ‘Idared’ apple peels when ultrasonication was used to facilitate the extraction. Response surface plots indicated that 80% to 100% methanol and ultrasonication duration of 15 min was the most effective combination for extracting quercetin and its glycosides from de-hydrated apple peel. Increased recovery of flavonols with higher concentrations of methanol could be attributed to the relatively non-polar nature of the flavonol molecules examined [15]. The decrease in the recovery of quercetin, quercetin-3-*O*-galactoside, and quercetin-3-*O*-rhamnoside over time could be due to the hydrolysis of glycosides and degradation of the aglycone due to extended extraction period. The temperature of ultrasonic bath was maintained below 30 °C though the quercetin has been shown to be relatively stable during various cooking processes except blanching [26].

Acidification of solvent is commonly practice when all sub-classes of phenolics are co-extracted with the aim of stabilizing anthocyanins [15]. When HCl was added to the methanol for extraction of flavonol from ‘Idared’ apple peel, it was observed that the increasing concentration of HCl is directly proportional to the recovery of quercetin, but inversely proportional to the recovery of quercetin glycosides. The observation suggests that when 0.1% (v/v) or higher concentrations of HCl incorporated in extraction solvent, glycosides release the aglycone quercetin due to hydrolysis. Therefore, in the presence of HCl in the extraction solvent, over estimation of quercetin in tested plant materials can be occurred. Furthermore, when HCl was incorporated at 1% and 2%, the total concentration of quercetin and glycosides were significantly decreased. This suggests that degradation of quercetin could occur under these conditions and the newly formed products need to be identified. Since most flavonols occur naturally in the glycosylated form, the acidification of solvents is not appropriate in terms of determining the distribution and concentration of naturally occurring flavonols in plant tissues. Quercetin galactoside was the most abundant flavonol in dehydrated apple peels when extraction was carried without acidification of methanol. Quercetin rhamnoside and quercetin rutinoside were the two other major flavonols identified. This is in agreement with previous studies by [9,12,23,24].

## 4. Experimental

### 4.1. Materials and Chemicals

‘Idared’ apple peels were collected from a commercial apple pie processing manufacturer (Apple Valley Foods Inc., Kentville, NS, Canada). HPLC-grade methanol, acetonitrile and formic acid were purchased from Sigma-Aldrich (St. Louis, MO, USA). The liquid chromatography standards used for the study were obtained as follows: Quercetin-3-*O*-rhamnoside and quercetin-3-*O*-galactoside were from Indofine Chemical Company (Hillsborough, NJ, USA); quercetin was from Sigma-Aldrich (St. Louis, MO, USA); and quercetin-3-*O*-rutinoside was from ChromaDex (Santa Ana, CA, USA). Other solvents were obtained from Fisher Scientific (Ottawa, ON, Canada).

### 4.2. Sample Preparation and Flavonol Extraction

The apple peels were processed into a dry powder using a previously described procedure [23]. For the extraction of flavonol molecules using different solvents, dehydrated apple peel (0.5 g) was mixed with solvent (25 mL) in glass stoppered Erlenmeyer flasks (125 mL capacity). In the subsequent study, two extraction factors were examined: percentage aqueous methanol as the extraction solvent and the duration in the ultrasonic bath. For each treatment, the extraction solvent (50 mL) was used to extract the phenolic molecules from dehydrated apple peel (1 g) using glass stoppered Erlenmeyer flasks (125 mL capacity). In another study, several levels of acidification of methanol were examined for quercetin and quercetin glycoside extraction from dehydrated apple peel powder (1 g). The extraction solvent used in this experiment was 50 mL of methanol containing 0%, 0.01%, 0.1%, 1% and 2% (v/v) HCl.

For the comparison of various solvents, 100% water, methanol, acetone, ethyl acetate and chloroform were used and the apple peel powder-solvent mixture was vortexed well, placed in the ultrasonic bath of 20 kHz/1000 Watts (model 750D, VWR, West Chester, PA, USA) and exposed for 60 min (four times for 15 min each with 10 min interval in between). The temperature of the ultrasonic bath was between 20 to 28 °C. Three replicates of each solvent were prepared. In the second study, the levels of methanol used were 60%, 70%, 80%, 90% and 100% (v/v). Three replicates of each methanol concentration were prepared using deionized water. The apple peel powder and the extraction solvent were vortexed and the flasks were placed in the ultrasonic bath for 15 min intervals up to 75 min, with 10 min breaks for sampling. Samplings consisted of removing 400 µL of the solution, which was filtered (nylon 0.45 micron filters, Waters Co., Milford, MA, USA) before being placed into HPLC auto-sample vials with inserts for the liquid chromatography, tandem mass spectrometry (HPLC-MS/MS) analysis [23,24]. Only exception to this procedure was when acetone, ethyl acetate and chloroform were used as the solvent, 400 µL of the extract was taken in small test tubes (12 × 75 mm) and evaporated under nitrogen followed by re-dissolving in 100% methanol before used for HPLC-MS/MS analysis. For the acidification experiment, ultrasonication was carried out for 15 min.

### 4.3. Liquid Chromatography and Mass Spectrometry Analysis

The HPLC system consisted of a Waters Alliance 2695 Separations Module and the column used was a Phenomenex Luna C18 (150 mm × 2.1 mm, 5 µm) with a Waters X-Terra MS C18 guard column. Gradient elution was carried out with 0.1% formic acid in water (Solvent A) and 0.1% formic acid in acetonitrile (Solvent B) at a flow rate of 0.35 mL/min. A linear gradient profile was used with the following proportions of Solvent A applied at time t (min); (t, A%): (0, 94%), (9, 83.5%), (11.5, 83%), (14, 82.5%), (16, 82.5%), (18, 81.5%), (21, 80%), (29, 0%), (31, 94%), (40, 94%). MS/MS experiments were preformed on a Waters Micromass Quattro micro API triple quadrupole mass spectrometer controlled by the Masslynx V4.0 data analysis system (Micromass, Cary, CA, USA). Electrospray ionization in negative ion mode (ESI-) was used for the analysis of the quercetin molecules. In negative mode, the following tune settings were used: capillary voltage 3,000 V, nebulizer gas temperature (N_2_) 375 °C at a flow rate of 600 L/h. The MS settings were tuned for each individual molecule. Analytes were identified and quantified using multiple reactions monitoring (MRM) mode in comparison with standards (Table 3). In the MS/MS experiments, both quadrupoles were operated at unit resolution.

**Table 3:** Retention times and ion transitions for flavonols quantified in apple peel powder using HPLC-MS/MS using multiple reactions monitoring (MRM) mode.

Flavonol molecule	Retention Time (min)	Ion Transition (m/*z*)
quercetin-3- *O*-rutinoside	16.36	609 → 301
quercetin-3- *O*-galactoside	17.51	464 → 301
quercetin-3- *O*-rhamnoside	23.00	448 → 301
quercetin	27.16	301 → 151

### 4.4. Experimental Design and Statistical Analysis

The statistical design of the experiment examining the effect of methanol concentration and extraction time was a two factor factorial design with the response as mg per 100 g dry weight (DW) of each flavonol. After ensuring normality and constant variance of the data using the Anderson-Darling test and an examination of the residuals versus fits plot, a two-way analysis of variance was used to check for significance at α = 0.05 using SAS 9.1 (SAS Institute, San Jose, CA, USA). As well, response surface curves for the extraction of the selected flavonols were created using Sigma Plot 8.0 (Systat Software, Richmond, CA, USA). The solvent comparison experiment and the acidification experiment used a completely randomized design with three replicates respectively to examine the difference in recovery of the quercetin molecules. The recovery of flavonols was compared using an analysis of variance with Tukey’s means comparison using SAS 9.1.

## 5. Conclusions

This study reveals that 80% to 100% methanol combined with exposure to 15 min ultrasonication could serve as an efficient method for the extraction of flavonols from ‘Idared’ apple peels. Even though this work is focused on only one apple cultivar, it is highly possible that the same extraction procedure could be adopted for the recovery of flavonols from the peels of other apple cultivars and similar plant matrices. Further studies on the influence of other critical variables such as sample to solvent ratio, particle size etc on the efficiency of flavonol extraction process from apple peels might be useful for scale-up extractions of flavonol molecules.
